# Bacterial Communities Associated with the Pine Wilt Disease Insect Vector *Monochamus alternatus* (Coleoptera: Cerambycidae) during the Larvae and Pupae Stages

**DOI:** 10.3390/insects11060376

**Published:** 2020-06-17

**Authors:** Hongjian Chen, Dejun Hao, Zhiqiang Wei, Lujie Wang, Tao Lin

**Affiliations:** 1 Co-Innovation Center for Sustainable Forestry in Southern China, Nanjing Forestry University, Nanjing 210037, China; chenhongjian1203@163.com (H.C.); zhiqiangw0127@163.com (Z.W.); wlj_963@126.com (L.W.); lintaonjfu@163.com (T.L.); 2 College of Forestry, Nanjing Forestry University, Nanjing 210037, China

**Keywords:** *Monochamus alternatus*, intestinal bacterial structure, larvae and pupae stages, symbiotic bacteria, 16S rRNA, functions

## Abstract

*Monochamus alternatus* is an important insect pest in pine forests of southern China and the dispersing vector of the pine wood nematode, *Bursaphelenchus xylophilus*, which leads to pine wilt disease (PWD). Microbiome of *M. alternatus* may contribute to survival of larvae in the host pine trees. In order to investigate the intestinal bacterial structure of *M. alternatus* during the larvae and pupae stages in host trees, and infer the function of symbiotic bacteria, we used 16S rRNA gene Illumina sequencing to obtain and compare the bacterial community composition in the foregut, midgut, and hindgut of larvae, pupal intestines, larval galleries, and pupal chambers of *M. alternatus*. The diversity of the bacterial community in larval intestines and pupal intestines were similar, as well as was significantly greater in larval galleries and pupal chambers. Although there were differences in bacterial compositions in different samples, similar components were also found. Proteobacteria and Firmicutes were the two most dominant phyla in all samples, and genera *Enterobacter*, *Raoultella*, *Serratia*, *Lactococcus*, and *Pseudomonas* were dominant in both the intestinal samples and plant tissue samples. *Enterobacter* was the most abundant genus in larval intestines, and *Serratia* was dominant in pupal intestine. The functions of these dominant and specific bacteria were also predicted through metagenomic analyses. These bacteria may help *M. alternatus* degrade cellulose and pinene. The specific role of symbiotic bacteria in the infection cycle of PWD also warrants further study in the future.

## 1. Introduction

The sawyer pine beetle, *Monochamus alternatus* Hope, causes serious damage to pine forests in southern China and is regarded as a forest pest in Asia due to vectoring an invasive pathogenic nematode, *Bursaphelenchus xylophilus* (Steiner et Buhrer) Nickle, which is known to cause pine wilt disease (PWD) [1,2,3]. At the beginning of the 20th century, PWD was first detected in Japan and subsequently spread to other Asian countries including China and Korea [4]. Recently, PWD has been reported successively in Portugal, Spain and the Portuguese island of Madeira in Europe [5]. PWD is considered to be one of the most serious diseases affecting conifers in the world. Throughout the larval and pupal stages, *M. alternatus* grows and develops entirely in host trees. The 1st and 2nd instar larvae feed on the phloem and cambium, and later instars tunnel into the xylem forming a characteristic U-shaped gallery which terminates a few millimeters short of the cambial layer [6,7]. The last-instar larvae then pupate in the terminal end of the gallery in a chamber formed when the larvae pack the gallery with shredded wood during gallery construction [6,8]. In nature, the pupal stage lasts from 17 to 19 days, and during this period, *B. xylophilus* enter the pupal cell [6,9].

Insects are colonized by microorganisms on their exoskeleton, in the intestine and hemocoel, and even in cells [10], and the species and quantity of microorganisms are the most in the intestine [11]. The intestinal microorganisms in insects generally include protists, fungi, archaea, and bacteria. The majority of organisms in the intestines of most insect species are bacterial species [12]. The intestinal tract of insects is composed of the foregut, midgut, and hindgut, which are important components of the digestive system [13]. The foregut is often used for temporary storage of food, the midgut is the main site of digestion and absorption, and the hindgut has the function of absorbing water and excreting food residues. The functional structure and physicochemical conditions in the different intestinal segments of insects result in the distribution of different microbial communities [10,14,15]. For many insects, intestinal microorganisms are mainly beneficial, as they contribute to the host’s development, pathogen resistance, nutrition, and physiology [12]. Recent studies showed that intestinal flora participate in the regeneration of intestinal cells and promotion of systemic development in *Drosophila melanogaster* [16,17]. Intestinal microorganisms that protect insects from pathogens have been reported in wasps, bumble bees, and *Anopheles gambiae* [18,19,20,21]. For many phytophagous insects, intestinal microorganisms can help degrade cellulose that is difficult to digest directly [22], and weaken the damage caused by plant defensive substances, such as terpene, against host insects [23,24]. In Hemiptera and Blattaria, intestinal microorganisms play a role in the synthesis of essential amino acids and the fixation of nitrogen [25,26,27]. Therefore, investigating intestinal symbiotic bacteria is helpful in providing an insight into insect physiology and has guiding significance for the identification of new methods of insect pest control. However, most studies have relied on high-throughput 16S rRNA gene amplicon sequencing in bacteria, preferring to observe the composition of insect intestinal communities [12]. 

The species, quantity, distribution, and function of symbiotic bacteria in different insects are diverse [11]. Symbiotic bacteria associated with several *Monochamus* species, mainly *Monochamus galloprovincialis* in Europe and *M. alternatus* in East Asia, have been described [5,7,28,29,30,31]. Through 16S rRNA sequencing analysis, the bacterial community mainly composed of Enterobacteriales, Pseudomonadales, Vibrionales, and Oceanospirillales in the trachea of *M. galloprovincialis* and *M. alternatus* has been reported [30]. In the thorax and abdomen of *M. galloprovincialis*, the bacterial community is dominated by the genera *Serratia*, *Bacillus*, and *Janthinobacterium* [5]. To date, there are few studies focused on the intestinal bacteria of *M. alternatus*. The community structure of intestinal bacteria in *M. alternatus* among different larval instars [31] and differences in the composition of bacterial communities in the midgut and hindgut of larvae and adults of *M. alternatus* fed natural or artificial diets [7] have been described based on high-throughput pyrosequencing.

Although some studies on the intestinal bacteria of *M. alternatus* have been carried out, there are still many issues to be resolved. Up to now, there are no reports on the microbial composition of pupa intestine in *M. alternatus*, whether the microbial composition of the larva intestine is consistent with that of the pupa intestine, the relationship between intestinal bacteria and environmental bacteria, and the potential function of intestinal bacteria. Our research is devoted to investigate the intestinal bacteria of *M. alternatus* from the above aspects. In order to have a deeper understanding of the intestinal bacteria in *M. alternatus* during the larvae and pupae stages in host trees, we used 16S rRNA gene Illumina sequencing to observe and comprehensively compare the bacterial community composition in the foregut, midgut, and hindgut of larvae, pupal intestines, larval galleries, and pupal chambers of *M. alternatus*. Therefore, the differences of the bacterial composition in different intestinal segments of the larvae, whether the gut flora from larvae stage to pupae stage have changed, and whether the intestinal bacteria of *M. alternatus* were related to the bacterial community of the host environment could be investigated. Metagenomic predictions were also applied to help us explore the potential functions of symbiotic bacteria in *M. alternatus.* These were performed to infer if the intestinal bacteria could help *M. alternatus* degrade cellulose for nutrition or resist pine defense substances, such as pinene.

## 2. Materials and Methods 

### 2.1. Sample Collection and Dissections

Two batches of natural PWD infested *Pinus massoniana* trees (n = 10) were collected from Lianxi district, Jiujiang City, Jiangxi Province, China (29°68′ N, 115°98′ E), in October and December 2016. The trees with PWD in each of the two batches were cut into logs 2 m in length and placed in cages covered with stainless steel wire mesh (4 mm^2^), with the following dimensions: 2.5 × 2.5 × 2 m. The first batch of preserved logs were cut with an axe in the direction of growth, sterile fine-tipped forceps were used to pick similar sized healthy *M. alternatus* larvae from the xylem galleries, and each larva was transferred into a 5 mL sterile centrifuge tube. Using sterile fine-tipped forceps, tissues from the larval galleries where the larvae were obtained were also transferred into 5 mL sterile centrifuge tubes. The second batch of preserved logs were cut in the same way as above, sterile fine-tipped forceps were used to pick similar sized healthy *M. alternatus* pupae from the xylem chambers, and each pupa was transferred into a 5 mL sterile centrifuge tube. Using sterile fine-tipped forceps, tissues from the pupal chambers where the pupae were obtained were also transferred into 5 mL sterile centrifuge tubes. All samples were stored at 4 °C and processed within 24 h of collection.

*M. alternatus* larvae (n = 30; represented by F) and pupae (n = 15; represented by FP) were surface sterilized with 70% ethanol for 1 min and rinsed twice with sterile water before dissection. Insects were dissected in 10 mM sterile phosphate-buffered saline (PBS) under aseptic conditions using dissection scissors and fine-tipped forceps. The head and last abdominal segment of each larva and pupa were severed, and pressure was applied anterior to the crop to release the gut. Each larval gut was divided into the foregut, midgut, and hindgut (represented by FF, FM, and FH), while the pupal guts were left intact. The guts were washed in PBS and either pooled or transferred individually into 1.5 mL microfuge tubes containing 0.1 mL or 0.5 mL of PBS. Every 10 segments of larval guts or 5 pupal guts were put into a microfuge tube, and three replicates were adopted. Guts in the tubes were sonicated (50/60 Hz, 117 V, 1.0 A; Branson Ultrasonics, Danbury, CT, USA) for 30 s, macerated with a plastic pestle, and vortexed at medium speed for 10 s to separate bacterial cells from the gut wall [32]. The tubes were then centrifuged at low speed (1000 rpm) and the supernatant collected for bacterial DNA extraction.

Larval gallery tissues (n = 15; represented by Z) and pupal chamber tissues (n = 15; represented by Y) were pooled and transferred into 1.5 mL microfuge tubes containing 0.5 mL of PBS. Each microfuge tube contained 5 Z or Y, and three replicates were adopted. Tissues in the tubes were sonicated (50/60 Hz, 117 V, 1.0 A; Branson Ultrasonics) for 30 s, macerated with a plastic pestle, and vortexed at medium speed for 10 s to separate bacterial cells from the tissues. The tubes were then centrifuged at low speed (1000 rpm) and the supernatant collected for bacterial DNA extraction.

### 2.2. DNA Extraction and PCR Amplification

DNA from samples was extracted using the hexadecyl trimethyl ammonium bromide (CTAB) method [33,34]. DNA concentration was assessed by a Nanodrop 2000C (Thermo Scientific, Waltham, MA, USA), and purity was monitored by 1% agarose gel electrophoresis. According to the concentration, DNA was diluted to 1 ng/μL using sterile water.

The V3-V4 regions of the bacterial 16S rRNA gene were PCR amplified using the following primers: 341 F (5′-CCTAYGGGRBGCASCAG-3′) and 806 R (5′-GGACTACNNGGGTATCTAAT-3′). All PCR reactions were carried out in 30 μL reactions with 15 μL of Phusion^®^ High-Fidelity PCR Master Mix (New England Biolabs, Ipswich, MA, USA), 0.2 μM of forward and reverse primers, and 10 ng template DNA. The reaction conditions were as follows: initial denaturation at 98 °C for 1 min, followed by 30 cycles of denaturation at 98 °C for 10 s, annealing at 50 °C for 30 s, elongation at 72 °C for 30 s, and finally 72 °C for 5 min. The same volume of 1× loading buffer (containing SYBR green, Thermo Scientific, Waltham, MA, USA) was mixed with PCR products, detected by 2% agarose gel electrophoresis, and further purified using the GeneJET Gel Extraction Kit (Thermo Scientific).

### 2.3. Gene Library Construction and Sequencing

Sequencing libraries were generated using the NEB Next^®^ Ultra^TM^ DNA Library Prep Kit for Illumina (NEB, Ipswich, MA, USA) following the manufacturer’s recommendations and index codes were added. The library quality was assessed by the Qubit^@^ 2.0 Fluorometer (Thermo Scientific) and Agilent Bioanalyzer 2100 system. The library was then sequenced on an Illumina HiSeq platform, and 250 bp paired-end reads were generated.

### 2.4. Data Analysis

Raw fastq files were quality-filtered by Trimmomatic and merged by FLASH [35] with the following criteria: (i) The reads were truncated at any site receiving an average quality score < 20 over a 50 bp sliding window. (ii) Sequences in which overlap being longer than 10 bp were merged according to their overlap with mismatch no more than 2 bp. (iii) Sequences of each sample were separated according to barcodes (exactly matching) and Primers (allowing 2 nucleotide mismatching), and reads containing ambiguous bases were removed.

Operational taxonomic units (OTUs) were clustered with 97% similarity cutoff using USEARCH (version 7.1 http://drive5.com/uparse/) with a novel ‘greedy’ algorithm that performs chimera filtering and OTU clustering simultaneously [36]. Only OTUs with ≥5 reads in at least three samples were retained. The taxonomy of each 16S rRNA gene sequence was analyzed by RDP Classifier algorithm (http://rdp.cme.msu.edu/) [37] against the Silva (SSU123) 16S rRNA database.

In order to compute alpha diversity, we rarified the OTU table and calculated three metrics: Sobs indicates the amount of unique OTUs found in each sample, Chao (Chao1) estimates species richness and Simpson describes community diversity. Significant differences in richness and diversity between two groups were evaluated using Student’s *t*-test when data had a normal distribution and Welch’s *t*-test when data did not satisfy the normal distribution assumption of ANOVA, and Pvalue was corrected with False Discovery Rate (FDR). SPSS Statistics (Armonk, USA) V22.0 was used to test the normal distribution of data. Rarefaction curves were generated by calculating the Sobs index under different random sampling using mothur.

We used unweighted unifrac for Principal Coordinates Analysis (PCoA), which showed beta diversity. It took a transformation from a distance matrix to a new set of orthogonal axes, and the maximum variation factor was demonstrated by the first principal coordinate, and the second maximum variation factor by the second principal coordinate, and so on. PERMANOVA was performed based on 999 permutations to test for significant differences.

Species and community composition analyses are represented by Venn and Bar plots. The Venn plot can be used to count the number of common and unique species (such as OTU) in multiple samples, and can visually show the similarity of species composition in environmental samples. The OTU table with 97% similarity level was selected for analysis. According to the Bar plot of the community, two aspects of information can be presented intuitively: (1) what types of microorganisms are contained in each sample at the taxonomic level; (2) the relative abundance of each microorganism in the sample, to understand the composition of the community structure of different samples at each taxonomic level. In order to identify the microbial communities with significant differences in relative abundance among the samples, Kruskal-Wallis H test and Scheffe post hoc tests was used to evaluate the significance level.

The dominant microbial communities in different samples can be represented by composite graphs of phylogenetic trees and reads abundance. Phylogenetic trees based on maximum likelihood of 16S rRNA gene sequences were constructed using FastTree (version 2.1.3 http://microbesonline.org/fasttree/). All the above analyses were performed with the statistical software R (version 3.5.1).

Phylogenetic Investigation of Communities by Reconstruction of Unobserved States (PICRUSt) was used to explore functional profiles [38]. The OTU table was standardized by PICRUSt to remove the effect of 16S marker gene on the number of copies in the genome of species. Then, through the Greengene ID corresponding to each OTU, we obtained the COG (Clusters of Orthologous Groups) family information and KEGG (Kyoto Encyclopedia of Genes and Genomes) information of OTU. According to the information of COG database, the description and function of each COG can be annotated, so as to obtain the function abundance spectrum. According to the KEGG database, information of KO (KEGG Orthology), pathway and EC (Enzyme) can be obtained, and the abundance of each functional category can be calculated according to the abundance of OTU. In addition, for pathway, three levels of metabolic pathway information can be obtained by PICRUSt, and the abundance tables of each level can be obtained, respectively. To evaluate the accuracy of metagenome predictions, the Nearest Sequenced Taxon Index (NSTI) was calculated, and lower values near zero indicate a closer relationship [38].

The raw data generated in this study can be found on NCBI, BioProject accession number PRJNA560442.

## 3. Results

### 3.1. Illumina Data

**Figure 1:**
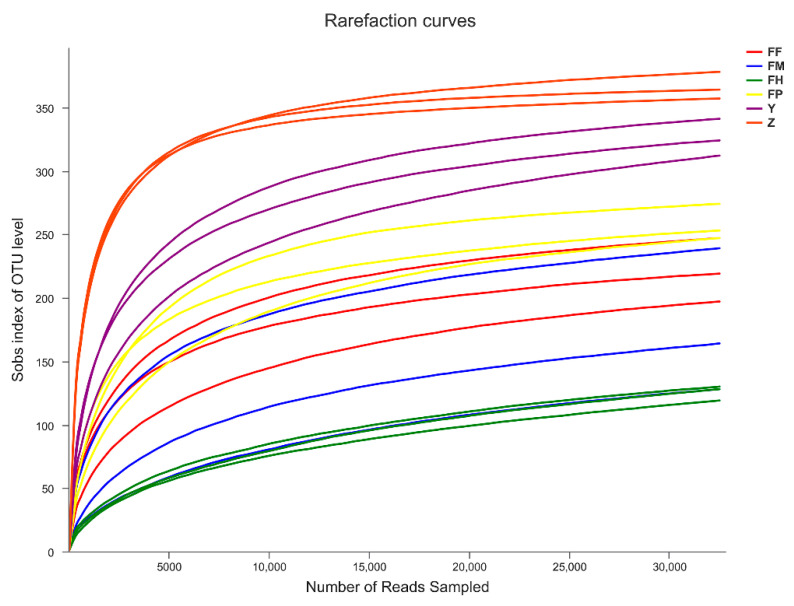
Rarefaction curves of bacterial communities in the intestine samples of *Monochamus alternatus* and tissue samples of *Pinus massoniana*. Sobs: number of species observed in the sample; FF: larval foreguts; FM: larval midguts; FH: larval hindguts; FP: pupal guts; Y: pupal chamber tissues; and Z: larval gallery tissues. All of these abbreviations apply to the following figures.

A total of 1,439,950 raw sequencing reads were obtained from Illumina HiSeq of 16S rRNA gene amplicons from 18 samples. After quality filtering and chimera removal, 999,437 (69.4%) high quality reads remained for analyses, with an average read length of 424 bp, ranging from 39,383 to 74,084 reads per sample. After subsampling the reads to an equal sequencing depth (32,485 sequences) between samples, a total of 606 OTUs were clustered at 97% sequence identity (Supplementary Table S1). The rarefaction curves suggested that all samples tended to saturation (Figure 1) and the Good’s coverage of each sample was above 99%, which indicated a sufficient depth of sequencing and capture of most bacterial diversity.

### 3.2. Alpha Diversity and Beta Diversity Analyses

**Figure 2:**
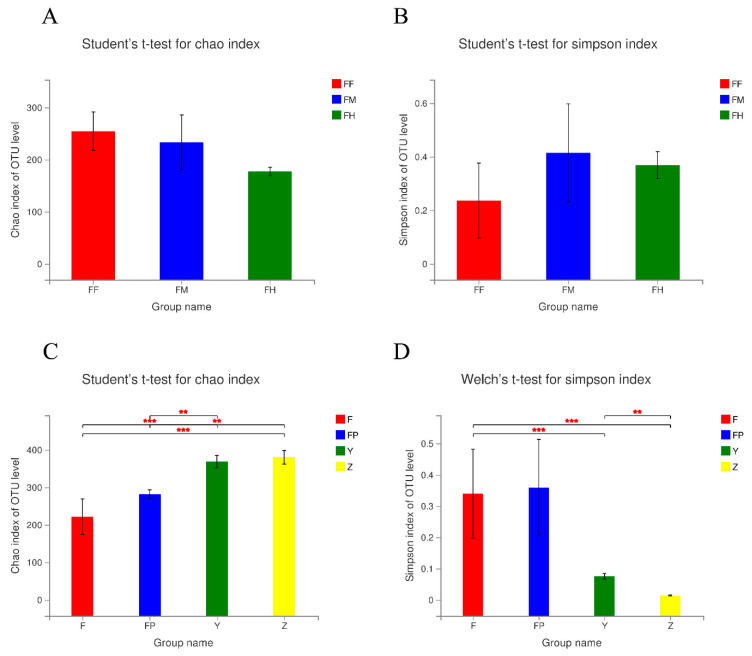
Alpha-diversity of bacterial communities in intestine samples of *M. alternatus* and tissue samples of *P. massoniana*. Significant differences in Chao (richness estimator) (**A**) and Simpson (diversity estimator) (**B**) indices in the three intestinal segments of larvae. Significant differences between all intestine samples and tissue samples in the Chao (**C**) and Simpson (**D**) index. F: larval guts (Student’s *t*-test, Welch’s *t*-test; * *p* < 0.05, ** *p* < 0.01, *** *p* < 0.001).

The alpha diversity of samples at the OTU level was estimated by the Chao and Simpson indices (Figure 2; Pvalue and FDR value are shown in Supplementary Table S2). We did not find significant differences in Chao index of FF, FM, and FH (Student’s *t*-test, *p* > 0.05; Figure 2A). The Simpson index also showed no significant differences between any two groups (Student’s *t*-test, *p* > 0.05; Figure 2B). By contrast, the Chao and Simpson indices of F, FP, Y, and Z were significantly different (*p* < 0.01; Figure 2C,D). The Chao index of Y and Z were both significantly higher than F and FP (Student’s *t*-test, *p* < 0.01), and the Simpson index of Y and Z were both significantly lower than F (Welch’s *t*-test, *p* < 0.001). The alpha diversity of these groups suggested that species richness and community diversity of microorganisms in the foregut, midgut, and hindgut were similar, but both were higher in the larval galleries and pupal chambers than in the intestines of larvae and pupae.

**Figure 3:**
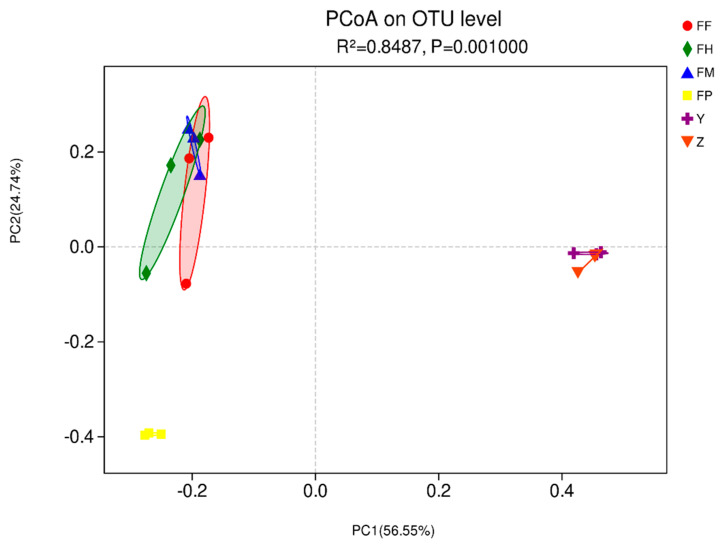
Beta-diversity of microbial communities in intestine samples of *M. alternatus* and tissue samples of *P. massoniana*. Principal Coordinates Analysis based on unweighted unifrac distances generated from operational taxonomic units (out) tables. Ellipses of different colors represent different groupings. (PERMANOVA; *p* = 0.001).

The beta diversity of samples at the OTU level described the similarities and differences of species composition and community structure. According to PCoA analysis (Figure 3), the microbial communities in all samples were divided into three clear groups (PERMANOVA, Df = 5, *R*^2^ = 0.8487, *p* = 0.001). We found that group FF, FM, and FH were clustered, group Y and Z gathered together, and FP was an independent group. These results showed that the foregut, midgut and hindgut shared similar microbial compositions, which were also similar in larval galleries and pupal chambers. However, the pupae differed in microbial composition with other samples.

### 3.3. Distribution of OTUs in Different Sample Groups

**Figure 4:**
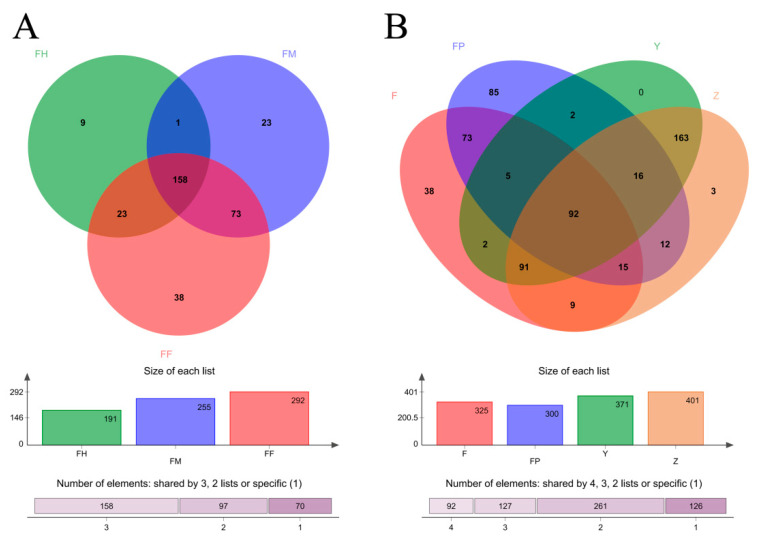
Venn diagrams of shared OTUs among different sample groups. (**A**) Venn diagrams of shared OTUs among the three intestinal segments of larvae. (**B**) Venn diagrams of shared OTUs among larval guts, pupal guts, pupal chamber tissues, and larval gallery tissues.

In order to investigate the similarity of microbial composition in different sample groups (different intestinal segments of larvae, larvae and pupae intestines, larval galleries, and pupal chambers), we used Venn diagrams at the OTU level (Figure 4). By comparing FF, FM, and FH, 158 OTUs were shared between three groups, and 97 OTUs were shared between two groups. Thirty-eight OTUs were unique to FF, 23 OTUs were found only in FM, and 9 OTUs were unique to FH. Thus, the three intestinal segments of larvae were similar in microbial composition, and the number of microbial OTUs increased from the hindgut to the foregut (Figure 4A). We also compared the OTUs in F, FP, Y, and Z (Figure 4B). 362 OTUs were found in both Y and Z, and only 48 OTUs were independently present in Y or Z; therefore, the microbial composition of larval galleries and pupal chambers was highly similar. In addition, 219 OTUs were shared among more than two groups, which were presumed to be microorganisms ingested into the intestine by larvae. Moreover, 185 OTUs were shared by F and FP, but 73 of these OTUs existed in F and FP alone. We reasoned that these 73 microbial OTUs colonized the intestine of *M. alternatus* over a long period of time. 

### 3.4. Microbial Community Composition of Samples at Different Taxonomic Levels

**Figure 5:**
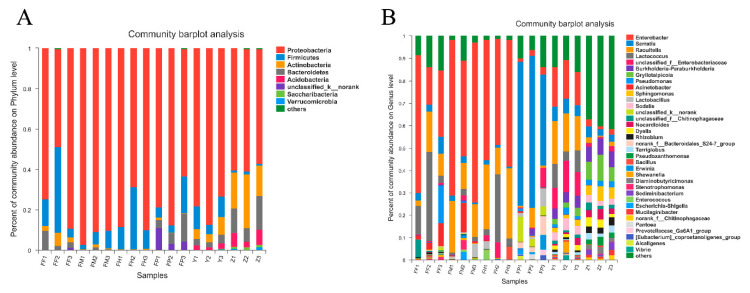
Relative abundance of microbial phyla and genera in intestine samples of *M. alternatus* and tissue samples of *P. massoniana*. Relative abundance of dominant microbial phyla (**A**; abundance ≥ 1%) and genera (**B**; abundance ≥ 2%) in larval foreguts, midguts, hindguts, pupal guts, pupal chamber tissues, and larval gallery tissues.

We analyzed the community composition of each sample at the phylum level and genus level. The bar plots showed the percent of community abundance at different taxonomic levels, while species with abundance less than 1% at phylum level and 2% at genus level were represented by others (Figure 5). The four most abundant phyla in all intestinal samples were Proteobacteria, Firmicutes, Bacteroidetes, and Actinobacteria, which were similar in larval galleries and pupal chambers with three more abundant phyla: Acidobacteria, Saccharibacteria, and Verrucomicrobia (Figure 5A). Furthermore, the unclassified phyla were found more in pupal intestines. At the genus level, 35 genera with high abundance were detected in all samples (Figure 5B). We found that genera with an average abundance of more than 1% in larval intestines were *Enterobacter*, *Raoultella*, *Lactococcus*, *Acinetobacter*, *Serratia*, *Pseudomonas*, unclassified_f__Enterobacteriaceae, and *Lactobacillus* (Supplementary Figure S1)*,* and in pupal intestines were *Serratia*, unclassified_k__norank, norank_f__Bacteroidales_S24-7_group, *Lactobacillus*, *Raoultella*, unclassified_f__Enterobacteriaceae, *Enterobacter*, and *Erwinia* (Supplementary Figure S2). In addition, we identified 13 genera with an average abundance of more than 2% both in larval galleries and pupal chambers. These 13 genera were *Enterobacter*, *Raoultella*, unclassified_f__Enterobacteriaceae, *Burkholderia-Paraburkholderia*, *Gryllotalpicola*, *Serratia*, *Lactococcus*, *Sphingomonas*, *Sodalis*, *Nocardioides*, *Dyella*, and *Pseudomonas* (Supplementary Figure S3).

**Figure 6:**
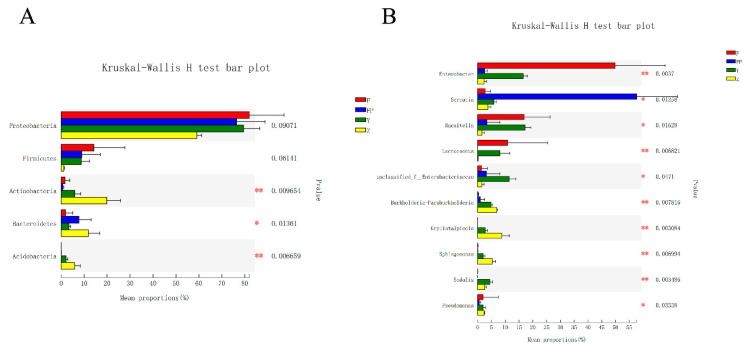
Differences in abundance of microbial phyla and genera in intestine samples of *M. alternatus* and tissue samples of *P. massoniana*. Differences in abundance of dominant microbial phyla ((**A**); top 5 are shown) and genera ((**B**); top 10 are shown) in larval guts, pupal guts, pupal chamber tissues, and larval gallery tissues. (Kruskal-Wallis H test; * *p* < 0.05, ** *p* < 0.01, *** *p* < 0.001).

We then identified all samples with different abundance at the phylum and genus levels, and tested the significance of the differences (Figure 6). Of the top 5 phyla with higher abundance, all sample groups had significant differences in abundance in 5 phyla except Proteobacteria and Firmicutes (Kruskal-Wallis H test, *p* < 0.05; Figure 6A). Then, of the top 10 genera with higher abundance, all genera showed a significantly different abundance in each sample group (Kruskal-Wallis H test, *p* < 0.05; Figure 6B). Preliminary results indicated that the abundance of *Enterobacter* (*p* = 0.004) was highest in larval intestines, while *Serratia* (*p* = 0.013) was richest in pupal intestines. Furthermore, the following four genera, *Burkholderia-Paraburkholderia* (*p* = 0.008), *Gryllotalpicola* (*p* = 0.003), *Sphingomonas* (*p* = 0.007), *and Sodalis* (*p* = 0.003)*,* showed higher abundance in larval galleries and pupal chambers than in intestinal samples.

### 3.5. Comparison of Dominant Microbial Communities in Different Sample Groups

**Figure 7:**
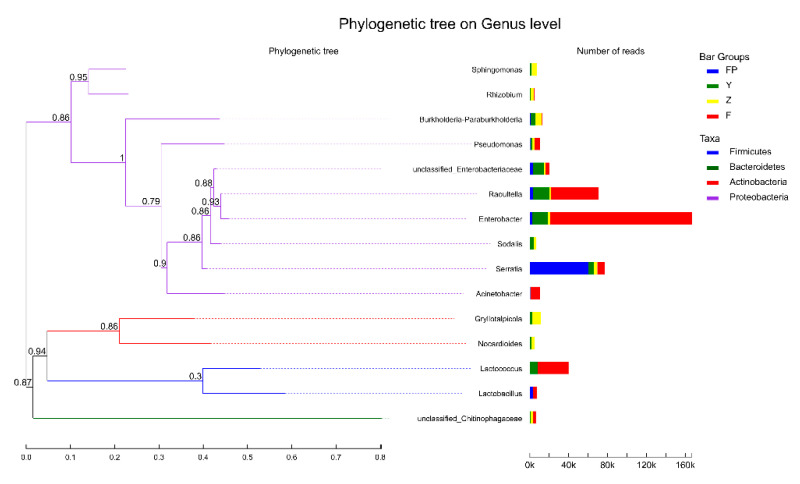
Phylogenetic trees based on maximum likelihood of 16S rRNA gene sequences of the top 15 genera in intestine samples of *M. alternatus* and tissue samples of *P. massoniana*. Phylogenetic trees of the top 15 genera of larval guts, pupal guts, pupal chamber tissues, and larval gallery tissues. The number of reads is represented by the length of the bar. The colors of the lines are representative of different phyla.

To determine the dominant microbial communities in intestinal samples, larval galleries, and pupal chambers, we constructed the phylogenetic trees of the top 15 genera (ranked by average abundance; Figure 7). *Enterobacter*, *Raoultella*, and *Lactococcus* were dominant genera in larval intestines, while *Serratia* was the dominant genus in pupal intestines. Proteobacteria was dominant in both larval and pupal intestines, and Firmicutes was dominant in larval intestines. In the remaining samples, *Gryllotalpicola*, *Burkholderia-Paraburkholderia*, and *Sphingomonas* were dominant in larval galleries, and *Enterobacter*, *Raoultella*, and unclassified_f__Enterobacteriaceae were dominant in pupal chambers. Proteobacteria and Firmicutes were the dominant phyla in pupal chambers, while Actinobacteria dominated in larval galleries in addition to Proteobacteria.

### 3.6. Functional Analysis of Metagenomes

**Figure 8:**
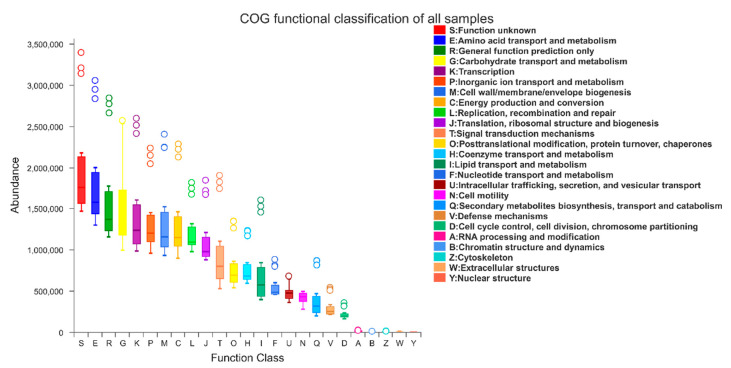
Clusters of Orthologous Groups (COG) functional classification of all samples. Level 2 COG functional annotation based on Phylogenetic Investigation of Communities by Reconstruction of Unobserved States (PICRUSt) of larval guts, pupal guts, pupal chamber tissues, and larval gallery tissues. The functions are represented by different colors.

Metagenomic inference of larval and pupal intestines, larval galleries, and pupal chambers was applied. The mean NSTI values were 0.090 ± 0.021 for larval intestines, 0.065 ± 0.040 for pupal intestines, 0.069 ± 0.002 for larval galleries, and 0.071 ± 0.003 for pupal chambers, indicating the high accuracy of metagenome predictions. A total of 24 Level 2 COG functions were annotated, except Nuclear structure (Y), and there were 9 functions with the median abundance higher than 1,000,000 (Figure 8). In addition to the unknown and general functions, Transport and metabolism of Amino acid (E) and Carbohydrate (G) were the functions with the highest predicted abundance. Functions, such as Intracellular trafficking, secretion, and vesicular transport (U); Secondary metabolites biosynthesis, transport, and catabolism (Q); and Defense mechanisms (V), which may be involved in nitrogen fixation, degradation of cellulose, and plant secondary defense substances, were also predicted.

In the functional annotations of KEGG, 293 Level 3 KEGG pathways of all samples were predicted (Supplementary Table S3), and 278 pathways were predicted for larval and pupal intestines, as well as 289 for larval galleries and pupal chambers. Both in the intestinal samples and host tissue samples, the function of cellulose and pinene degradation according to Starch and Sucrose Metabolism (Supplementary Figure S4: intestinal samples, S5: host tissue samples, corresponding to KEGG pathway map00500), and Limonene and Pinene Degradation (Supplementary Figure S6: intestinal samples, S7: host tissue samples, corresponding to KEGG pathway map00903) pathway was predicted. For larval and pupal intestines, an average of 132,349 ± 33,957 Starch and Sucrose Metabolism abundance, 46,296 ± 14,132 Limonene and Pinene Degradation abundance were predicted, 218,572 ± 56,593 and 108,945 ± 44,830 for larval galleries and pupal chambers. Cellulase/endoglucanase (EC: 3.2.1.4), cellulose 1,4-beta-cellobiosidase (EC: 3.2.1.91), beta-glucosidase (EC: 3.2.1.21), and cellobiose phosphorylase (EC: 2.4.1.20) involved in the degradation of cellulose were annotated. Alpha-pinene dehydrogenase/monooxygenase (EC: 1.14.-.-), enoyl-CoA hydratase (EC: 4.2.1.17), limonene 1,2-monooxygenase (EC: 1.14.13.107), limonene-1,2-epoxide hydrolase (EC: 3.3.2.8), (+)-trans-carveol dehydrogenase (EC: 1.1.1.275), and aldehyde dehydrogenase (EC: 1.2.1.3), which participate in the degradation of limonene and pinene, were also annotated.

## 4. Discussion

Our research based on high-throughput sequencing adds to the understanding of intestinal bacteria in *M. alternatus*, and some interesting results were obtained. The bacterial composition and diversity of different intestinal segments of larvae were similar, but the intestinal bacterial composition between larvae and pupae were different. On the other hand, some of the intestinal bacteria in *M. alternatus* were the same as those in the larval galleries and pupal chambers. The results of metagenomic inference showed that some intestinal symbiotic bacteria played an important role in digesting lignocellulose to obtain nutrients and degrade terpenes toxic to beetles secreted by conifers when *M. alternatus* fed on host pine trees.

In different intestinal segments of larvae, the diversity of intestinal microbial communities was similar. The microbial abundances in the foregut exceeded that of other intestinal segments due to the short-term storage of food, which has also been found in other insects [10]. We also found no significant difference in the richness and diversity of intestinal bacteria between larvae and pupae, but there were differences in species composition and structure. This phenomenon could be explained by remodeling of the gut and other organs during metamorphosis, and the removal of intestinal bacteria, which are wrapped in the peritrophic matrix of pupae as meconium, from larvae [12,39]. By contrast, larval galleries and pupal chambers had significantly higher microbial richness and diversity. This may indicate that the living environment of *M. alternatus* had more complex and diverse microbial communities than the intestines. In the galleries initiated by *Dendroctonus valens* (Coleoptera: Curculionidae), much higher densities of microorganisms have been detected than in non-infested red pine subcortical tissues [40]. From the results of beta diversity, it can be seen that the microbial composition of different intestinal segments in *M. alternatus* larvae was highly similar. The microorganisms in the midgut and hindgut of wild *M. alternatus* larvae showed the same consistency using detrended correspondence analysis [7]. Microorganisms in larvae, pupae, larval galleries, and pupal chambers formed unique communities, and there were some differences between these groups. The microbial composition of larval galleries and pupal chambers also showed similarity. Pupal intestines showed a significant difference in microbial communities compared with other groups, which confirmed that the structure of intestinal microorganisms was remodeled after pupation.

The distribution of OTUs showed that the foregut, midgut, and hindgut of larvae shared most of the intestinal bacteria, and the foregut had the largest number of symbiotic bacteria. This result was similar to that of alpha and beta diversity analyses. All three intestinal segments had their own unique species, with the largest number in the foregut and the smallest number in the hindgut. In insects, the foregut usually acts as a site for temporary food storage, thus creating more opportunities for colonization of environmental microorganisms [12]. Our results are similar to those for the intestinal bacterial community structure of soil-feeding termites [41]. During *M. alternatus* larvae feeding, microorganisms enter the intestine from the host environment, producing more transient populations in the foregut, which decreased in the midgut and hindgut in turn. As larval galleries and pupal chambers are connected in the xylem of *P. massoniana*, their microbial composition showed high similarity. In this study, the number of microbial species in the host environment of *M. alternatus* was higher than that in the intestines. Many identical microbial species were present in the intestinal tract of larvae and pupae, as well as in the larval galleries and pupal chambers. In *Scaphoideus titanus* (Hemiptera: Cicadellidae), flavescence dorée phytoplasma can be acquired from grapevines and multiplies in the foregut and midgut following feeding [42]. The midgut bacterial community of *Helicoverpa armigera* (Lepidoptera: Noctuidae) is similar to that of the leaf eaten by larvae [43]. The majority of *Ceratina* bees and their pollen were also found to share a similar microbial composition [44]. Our research and the above reports all indicate that the microbial flora of the intestine is affected by that of the host on which insects feed. The *M. alternatus* larvae and pupae intestines also have their unique long-term colonized microbiomes. 

Proteobacteria and Firmicutes were the two most dominant phyla in all samples. In previous studies on the intestinal bacteria of the larvae and adults of *M. alternatus*, the dominance of these two phyla was reported [7,31]. Proteobacteria and Firmicutes also predominated in other insects of Cerambycidae and some Diptera, such as *Bactrocera minax*, *Aedes aegypti*, and *Anastrepha* [5,30,32,45,46,47,48,49,50,51]. The high abundance of these phyla in insects may be due to their being more likely to invade and colonize insect hosts than other bacterial communities [52]. In addition, our results showed, for the first time, that Proteobacteria and Firmicutes were the dominant phyla in the larval galleries and pupal chambers of *M. alternatus*. By comparison, higher abundance of Saccharibacteria and Verrucomicrobia, which are common environmental bacteria in soil and water [53,54,55], were found in the larval galleries and pupal chambers rather than in the intestines. Saccharibacteria has the ability to degrade cellulose, hemicellulose, and pectin [56]. 

*Enterobacter*, *Serratia*, *Raoultella*, *Lactococcus*, *Acinetobacter*, *Lactobacillus*, *Pseudomonas*, and *Erwinia* were the major genera in larval and pupal intestines. The same dominant genera were found in the intestines of different instar larvae of *M. alternatus*, and some were also previously observed in the intestines of adult *M. alternatus* [7,31]. *Raoultella* and *Lactobacillus* were first reported as dominant genera related to *M. alternatus*. Most of these genera (*Enterobacter*, *Serratia*, *Raoultella*, *Erwinia*) were classified as Enterobacteriaceae, which was a constant fraction of the symbiotic intestinal communities from humans to insects, and usually contributed to vitamin biosynthesis, pheromone production, nitrogen fixation, and degradation of plant compounds [32,48,57,58]. Additionally, other common functions of these genera have been found in insect intestines. *Serratia marcescens* helps to consume oxygen in the intestine of the Formosan termite, thus, maintaining a habitable intestine for the strict anaerobic bacteria that digest cellulose [59]. It has been reported that *Pseudomonas, Serratia, Erwinia*, *Enterobacter*, and *Burkholderia* aid bark beetles in the metabolism of coniferous defense compounds, such as monoterpenes and diterpene acids [23,24,60,61,62]. It is believed that *Lactococcus lactis*, which was dormant on plant surfaces, proliferated in the intestines of insects after feeding [63]. Some other members of *Lactococcus* have been reported to be associated with fermentation and to assist longicorn beetles in the degradation of cellulose [12,64,65]. *Acinetobacter* was found to have lignin degradation activity in previous experiments [66]. *Lactobacillus* participated in sugar decomposition, as well as acid and bacteriocin production, to protect the intestinal environment from putrefactive and sulfate-reducing bacteria [67]. In addition to the same genera in larval and pupal intestines, some other genera with higher abundance in larval galleries and pupal chambers have also been detected, such as *Burkholderia-Paraburkholderia* and *Gryllotalpicola*. In a mason bee and a wood-feeding termite, *Burkholderia* and *Gryllotalpicola* were, respectively, observed [68,69]. We inferred that the tissues of larval galleries and pupal chambers were contaminated by longicorn feces. *Burkholderia* has been confirmed or predicted in previous studies to have nitrogen fixation ability, defense mechanisms, and can detoxify tree-defense compounds and degrade aromatic compounds [70]. 

*Lactococcus* and *Acinetobacter*, which was enriched in the intestinal samples, may contribute to cellulose degradation in the host [12,64,65,66]. *Pseudomonas* was also found in all samples. We speculate that terpenes may be degraded by *Pseudomonas* in the intestine of *M. alternatus* to resist further toxicity [23,24,60,61]. Functional prediction showed that the bacterial community of intestinal samples and host tissue samples are involved in the degradation of cellulose and pinene, and several key enzymes were annotated. These results preliminarily verified the possible functions of these genera when *M. alternatus* fed on *P. massoniana* trees.

Although larvae and pupae are two successive life stages of *M. alternatus*, their intestinal microbiota structure were not similar. The microbial structure in the intestines of *Agrilus planipennis* larvae and prepupae also showed dissimilarities [71]. *Serratia*, which was found in our study as the most abundant genus in pupal intestines, was previously reported as a major group associated with the pine wood nematode (PWN) [29]. The PWN was attracted to the pupal chambers during its dispersal stage, and immediately entered the pupal tracheae of *M. alternatus* [72]. Then, PWN was mainly transmitted by newly emerged adults, entering a healthy pine tree through feeding wounds made by *M. alternatus* [6]. The pine tree could die as a consequence of PWN infection. However, the feeding wounds can induce pine tree to release a large number of chemical defense substances, such as terpenoids [73]. Some symbiotic bacteria can enhance the virulence of the PWN by affecting the expression levels of genes related to cell wall degradation, detoxification, and reproduction [74]. We hypothesized that the PWN can capture bacteria from the pupae of *M. alternatus*, and these bacteria may help PWN effectively infect healthy pine trees. Although our study described the composition of intestinal bacteria in two successive life stages of *M. alternatus* and predicted the potential function of the dominant bacteria, the functional mechanism of these bacteria in the intestine of *M. alternatus* and whether they assist PWN in infecting pine trees still needs further study.

## Supplementary Materials

The following are available online at https://www.mdpi.com/2075-4450/11/6/376/s1, Figure S1: Community analysis pieplot on genus level of larval guts, Figure S2: Community analysis pieplot on genus level of pupal guts, Figure S3: Community analysis pieplot on genus level of pupal chamber tissues, and larval gallery tissues, Figure S4: KEGG pathway of Starch and Sucrose Metabolism mapped by intestinal samples, Figure S5: KEGG pathway of Starch and Sucrose Metabolism mapped by host tissue samples, Figure S6: KEGG pathway of Limonene and Pinene Degradation mapped by host tissue samples, Figure S7: KEGG pathway of Limonene and Pinene Degradation mapped by host tissue samples, Table S1: OTU species classification statistics, Table S2: Pvalue and FDR value of Chao, and Simpson indices, Table S3: KEGG pathway level 3 abundance of each sample.
