# Supplementary figures and images for: Bacterial Communities Associated with the Pine Wilt Disease Insect Vector Monochamus alternatus (Coleoptera: Cerambycidae) during the Larvae and Pupae Stages
Source: Insects. 2020 Jun 17;11(6):376. doi: 10.3390/insects11060376 (PMC7348839; doi:10.3390/insects11060376)

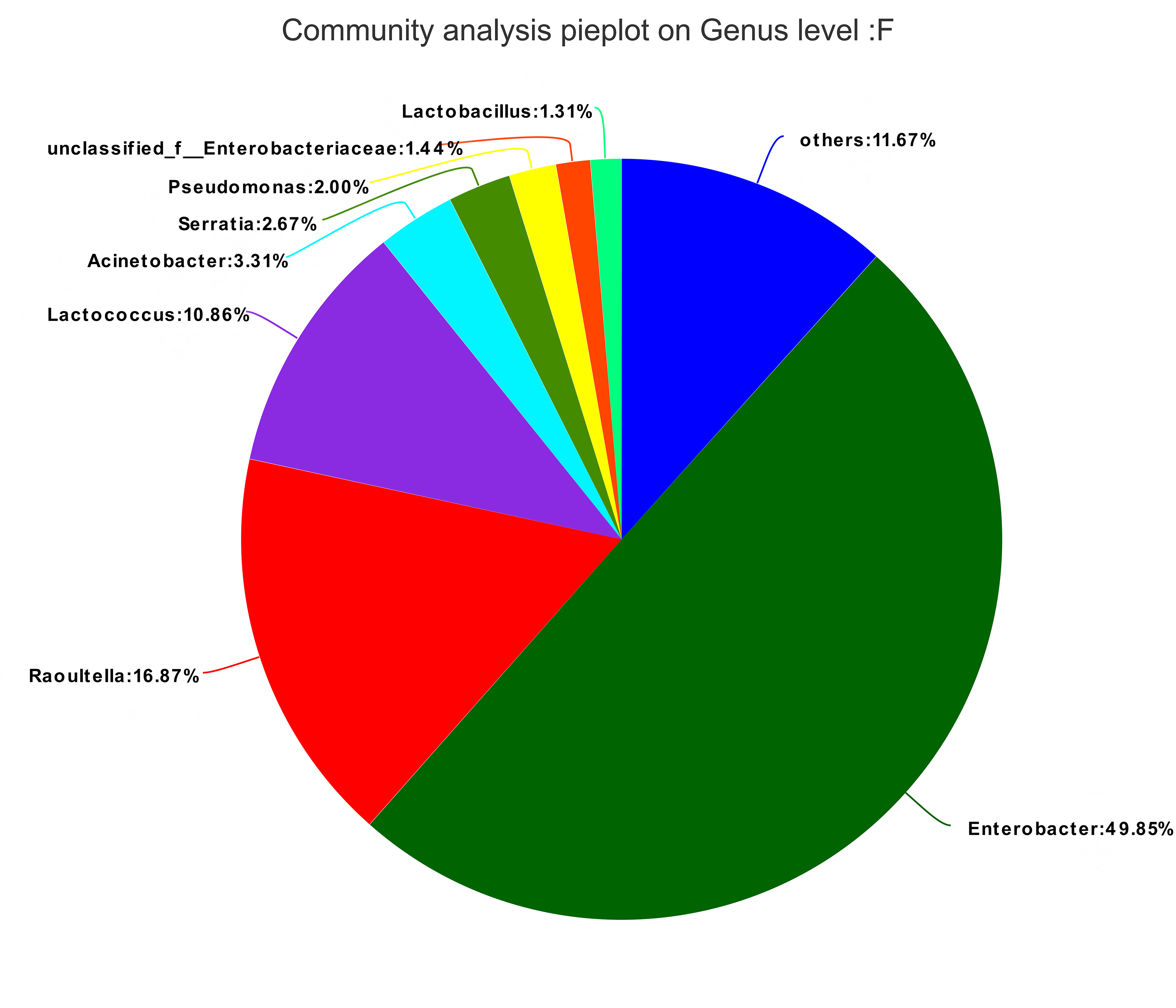

Supplement: Supplementary file 1 [file insects-11-00376-s001.zip › Supplementary Files/Supplementary Figure S1.tif]

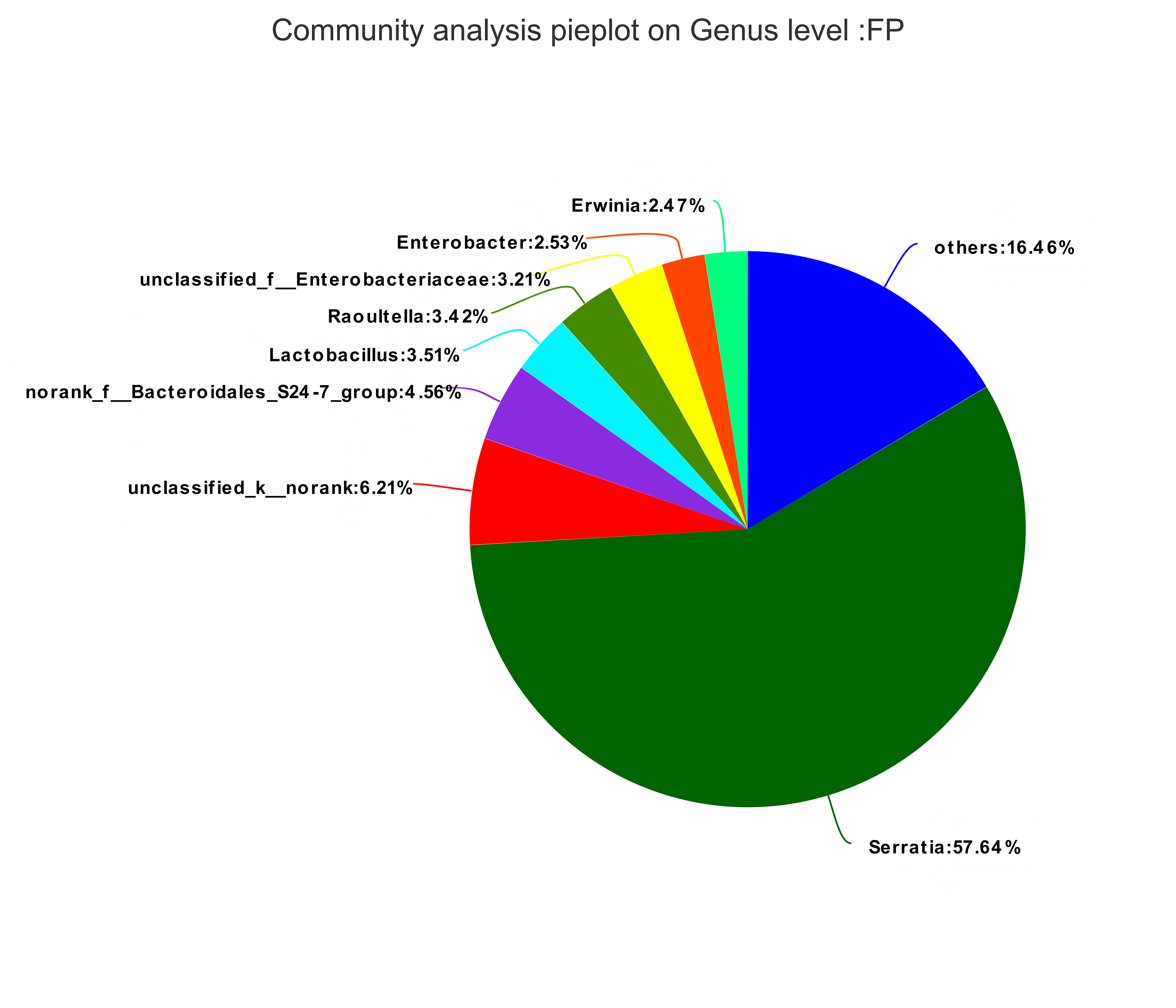

Supplement: Supplementary file 1 [file insects-11-00376-s001.zip › Supplementary Files/Supplementary Figure S2.tif]

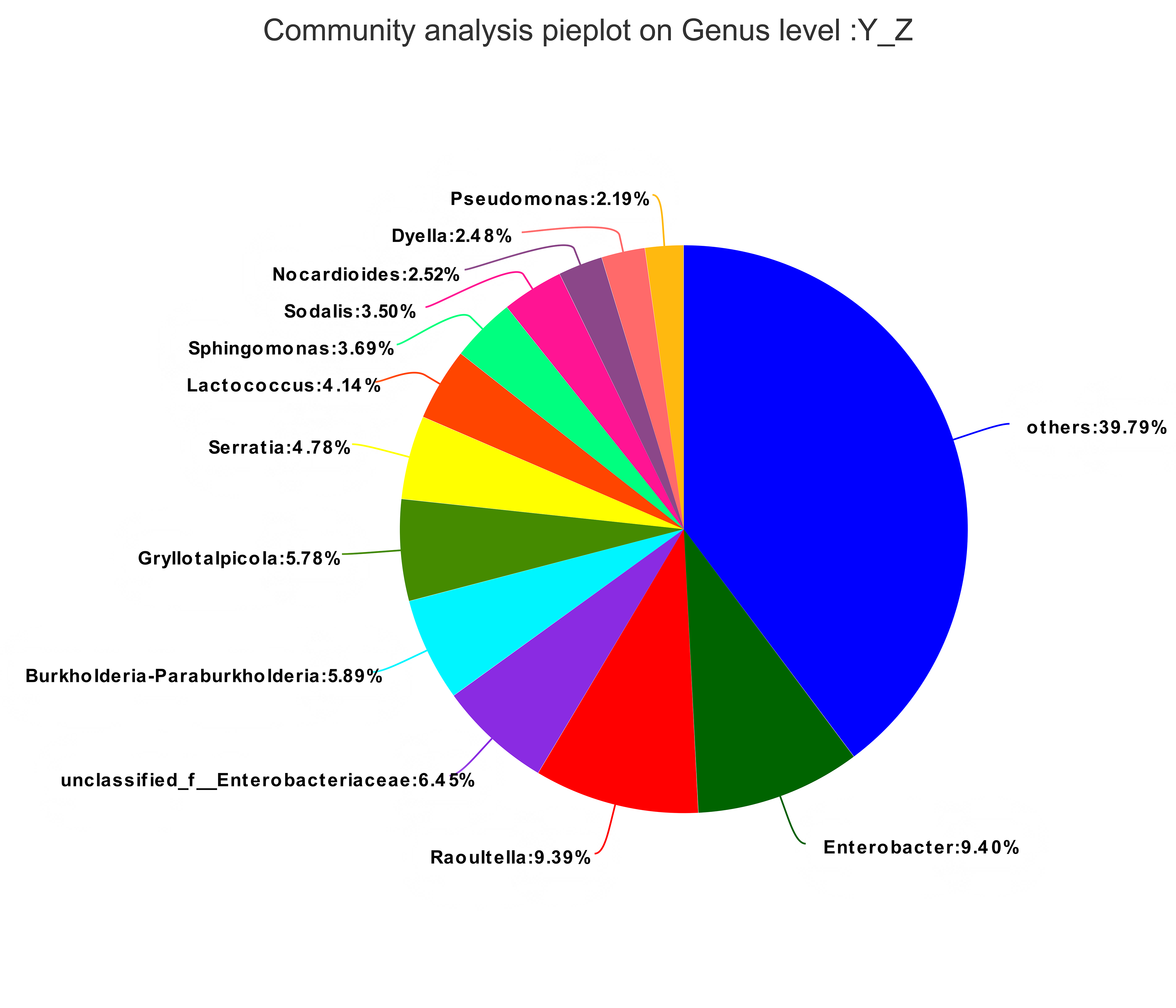

Supplement: Supplementary file 1 [file insects-11-00376-s001.zip › Supplementary Files/Supplementary Figure S3.tif]

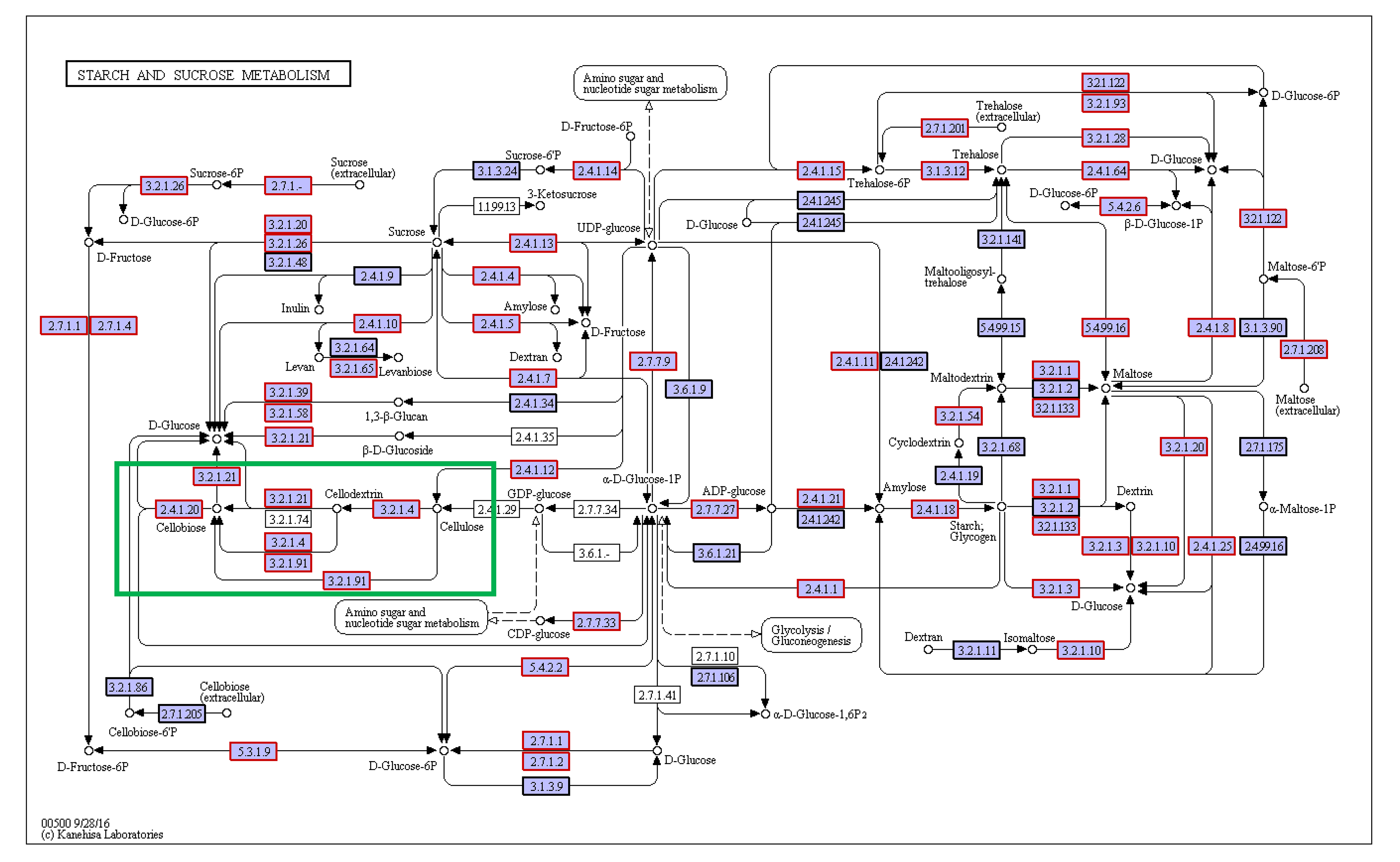

Supplement: Supplementary file 1 [file insects-11-00376-s001.zip › Supplementary Files/Supplementary Figure S4 ko00500 F-FP.tif]

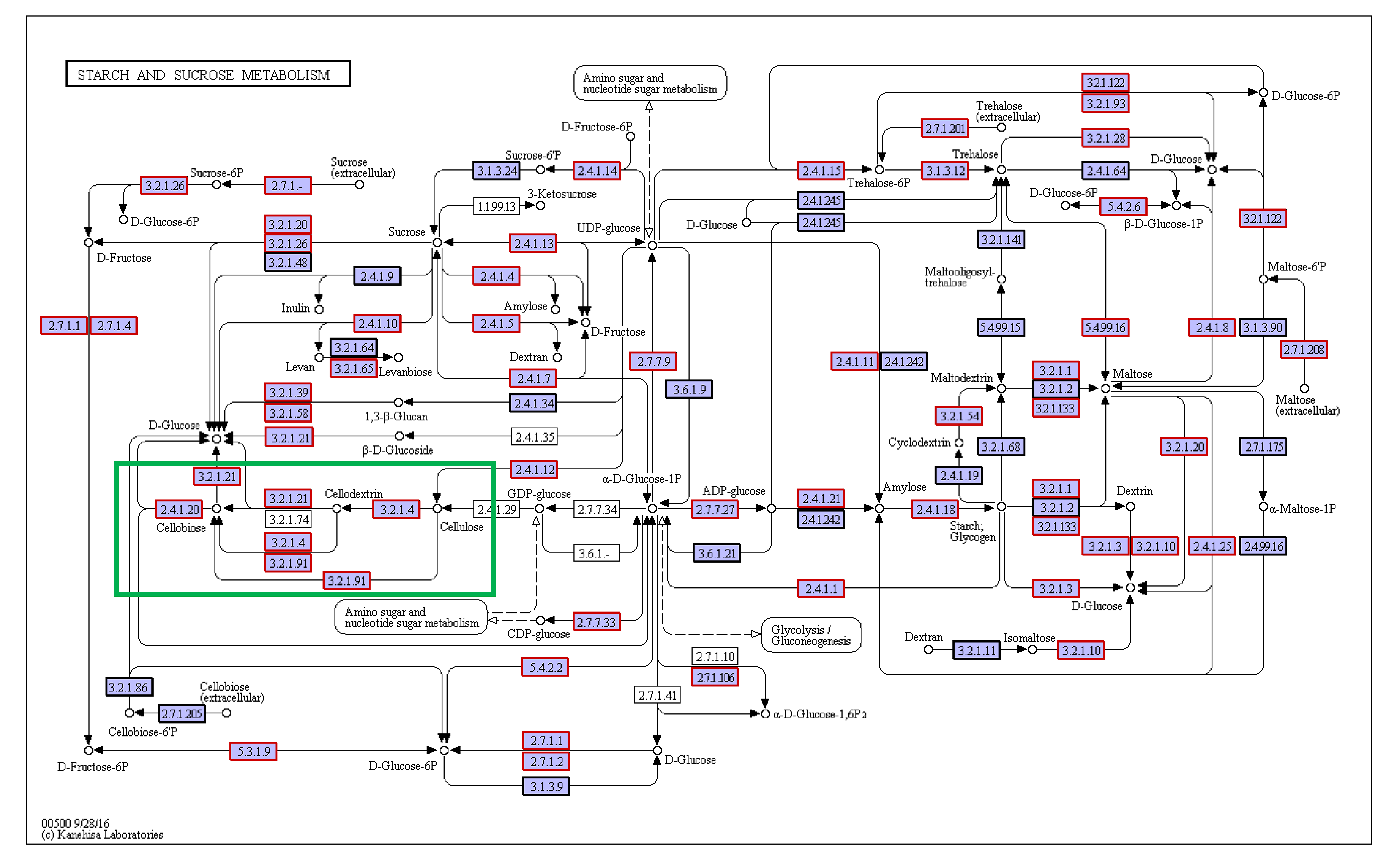

Supplement: Supplementary file 1 [file insects-11-00376-s001.zip › Supplementary Files/Supplementary Figure S5 ko00500 Y-Z.tif]

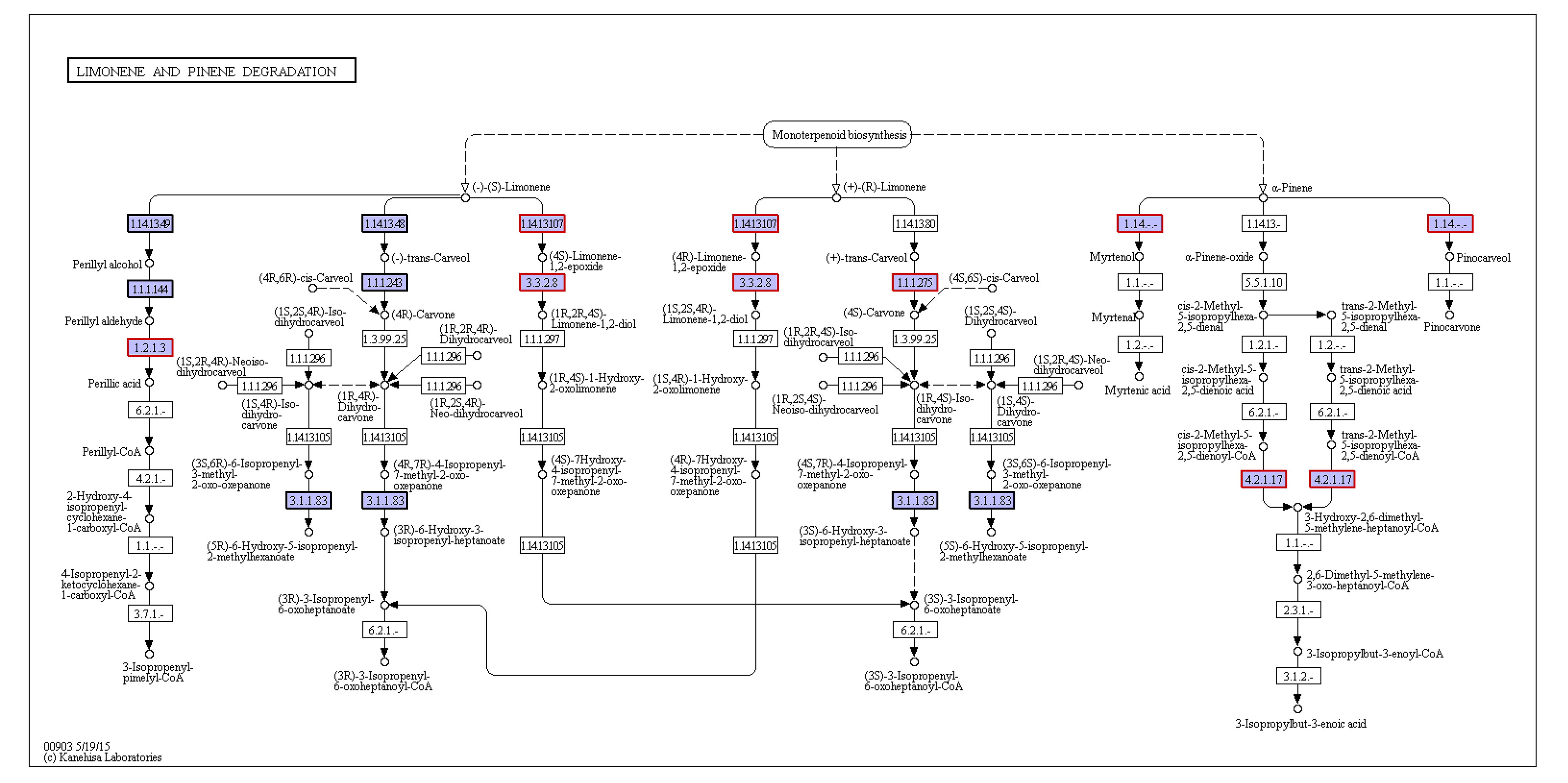

Supplement: Supplementary file 1 [file insects-11-00376-s001.zip › Supplementary Files/Supplementary Figure S6 ko00903 F-FP.tif]

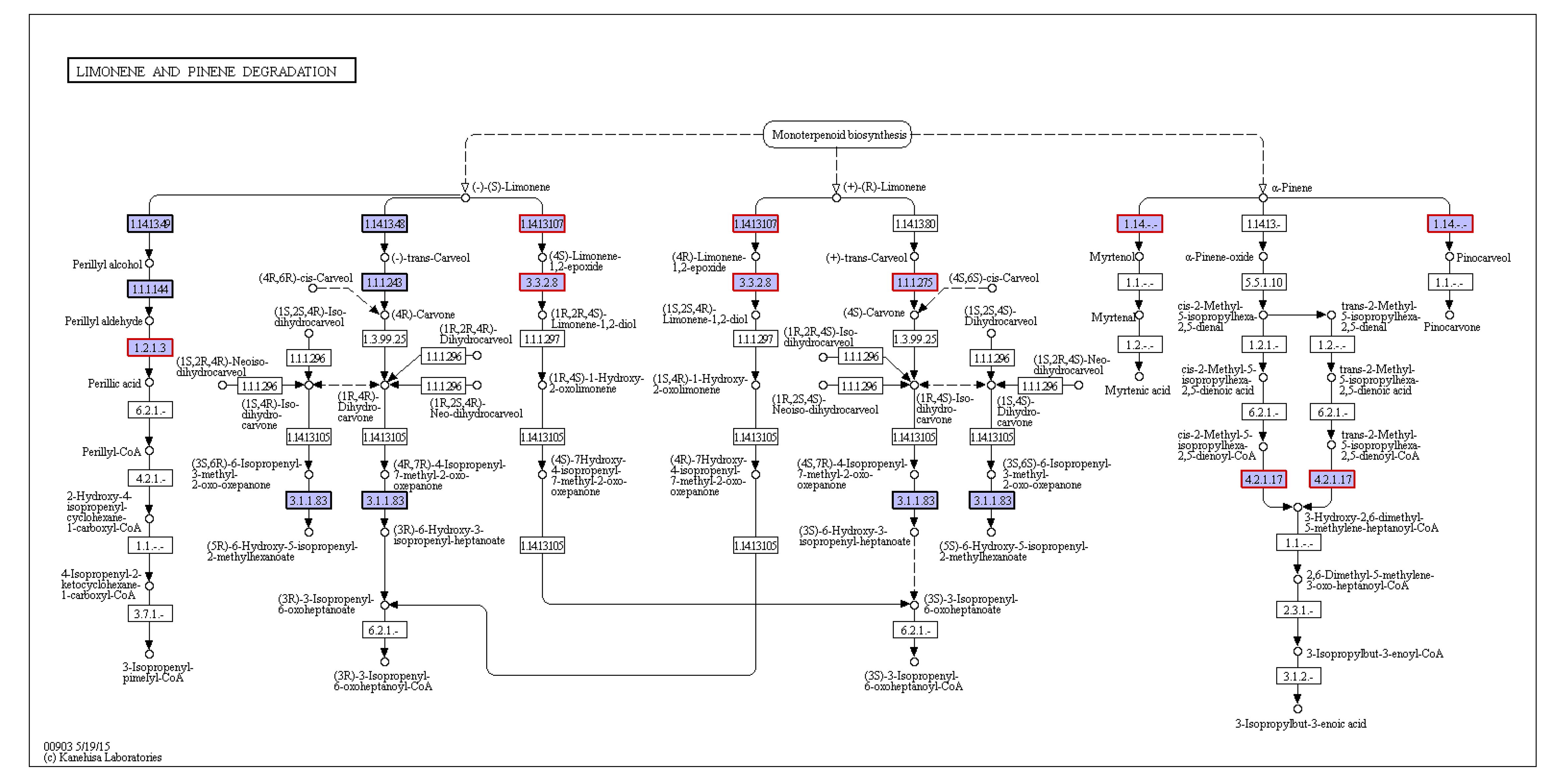

Supplement: Supplementary file 1 [file insects-11-00376-s001.zip › Supplementary Files/Supplementary Figure S7 ko00903 Y-Z.tif]
